# Process evaluation of a knowledge translation intervention using facilitation of local stakeholder groups to improve neonatal survival in the Quang Ninh province, Vietnam

**DOI:** 10.1186/s13063-015-1141-z

**Published:** 2016-01-13

**Authors:** Leif Eriksson, Tran Q. Huy, Duong M. Duc, Katarina Ekholm Selling, Dinh P. Hoa, Nguyen T. Thuy, Nguyen T. Nga, Lars-Åke Persson, Lars Wallin

**Affiliations:** International Maternal and Child Health, Department of Women’s and Children’s Health, Uppsala University, Uppsala, Sweden; Nursing office, Department of Medical Services Administration, Ministry of Health, Hanoi, Vietnam; Hanoi School of Public Health, Hanoi, Vietnam; Vietnam Sweden Uong Bi General Hospital, Quang Ninh, Vietnam; Department of Neurobiology, Care Sciences and Society, Division of Nursing, Karolinska Institutet, Stockholm, Sweden; School of Education, Health and Social Studies, Dalarna University, Falun, Sweden

**Keywords:** community health workers, facilitation, knowledge translation, neonatal health, neonatal mortality, process evaluation, Vietnam

## Abstract

**Background:**

Annually, 2.8 million neonatal deaths occur worldwide, despite the fact that three-quarters of them could be prevented if available evidence-based interventions were used. Facilitation of community groups has been recognized as a promising method to translate knowledge into practice. In northern Vietnam, the Neonatal Health – Knowledge Into Practice trial evaluated facilitation of community groups (2008–2011) and succeeded in reducing the neonatal mortality rate (adjusted odds ratio, 0.51; 95 % confidence interval 0.30–0.89). The aim of this paper is to report on the process (implementation and mechanism of impact) of this intervention.

**Methods:**

Process data were excerpted from diary information from meetings with facilitators and intervention groups, and from supervisor records of monthly meetings with facilitators. Data were analyzed using descriptive statistics. An evaluation including attributes and skills of facilitators (e.g., group management, communication, and commitment) was performed at the end of the intervention using a six-item instrument. Odds ratios were analyzed, adjusted for cluster randomization using general linear mixed models.

**Results:**

To ensure eight active facilitators over 3 years, 11 Women’s Union representatives were recruited and trained. Of the 44 intervention groups, composed of health staff and commune stakeholders, 43 completed their activities until the end of the study. In total, 95 % (*n* = 1508) of the intended monthly meetings with an intervention group and a facilitator were conducted. The overall attendance of intervention group members was 86 %. The groups identified 32 unique problems and implemented 39 unique actions. The identified problems targeted health issues concerning both women and neonates. Actions implemented were mainly communication activities. Communes supported by a group with a facilitator who was rated high on attributes and skills (*n* = 27) had lower odds of neonatal mortality (odds ratio, 0.37; 95 % confidence interval, 0.19–0.73) than control communes (n = 46).

**Conclusions:**

This evaluation identified several factors that might have influenced the outcomes of the trial: continuity of intervention groups’ work, adequate attributes and skills of facilitators, and targeting problems along a continuum of care. Such factors are important to consider in scaling-up efforts.

**Trial registration:**

ISRCTNISRCTN44599712.

**Electronic supplementary material:**

The online version of this article (doi:10.1186/s13063-015-1141-z) contains supplementary material, which is available to authorized users.

## Background

In efforts at reducing under-five-year-old mortality, it has become evident that both women’s and children’s health need to be focused on along a continuum of care [1]. A particularly important period is around the time of delivery. Currently, 2.8 million deaths occur worldwide during the neonatal period (the first 28 days after delivery), corresponding to 44 % of under-five-year-old deaths [2]. Fortunately, available evidence-based interventions exist that can help avoid three out of four neonatal deaths [3, 4]. The best method of implementation of these interventions in a sustainable way is still unclear.

Facilitation is a knowledge translation approach, whereby one person (the facilitator), using an active and dynamic working strategy, helps and enables a group of people through a developing and learning process [5]. A group working together with a facilitator could be viewed as a coalition, i.e., a partnership aiming at change and the introduction of innovative solutions to health problems [6]. Hence, facilitation is a team effort [7, 8]. Reviews of the facilitation method [5, 8, 9] unanimously conclude that it is a promising knowledge translation method. Even though some recent studies contribute to increased comprehension of the concept and role of facilitation [10–13], there is still a need of more knowledge from different contexts as to what attributes and skills a facilitator should possess, how to train and support facilitators and the impact of facilitation in knowledge translation [5, 8, 9, 11].

During the past decade or so, there has been an increased focus on knowledge translation interventions in low- and middle-income countries using empowerment and participation at the community level to increase neonatal survival [14–16]. In Nepal, a groundbreaking study was conducted in which facilitators targeted women’s groups [17]. In that study, women were facilitated to identify and formulate actions to address perinatal problems, resulting in a reduction in neonatal mortality rate of 30 % and increased coverage of antenatal care, institutional deliveries, skilled birth attendance and hygienic care. Replications of the Nepalese study in South Asia and Africa have also been successful [18, 19].However, the reduction in neonatal mortality is limited with this approach when trying to cover larger populations [20].

Inspired by the study in Nepal, our research team conducted the Neonatal Health – Knowledge Into Practice trial (NeoKIP, trial registration ISRCTN44599712) for 3 years in a northern province of Vietnam. This trial investigated the effectiveness of facilitation as a knowledge translation intervention for improving neonatal health and survival [21]. The intervention resulted in increased attendance to antenatal care clinics and reduced neonatal mortality (adjusted odds ratio 0.51; 95 % confidence interval, 0.30–0.89) after a latent period [22]. In contrast with the trials targeting women’s groups [18, 19], we decided to compose groups consisting of local healthcare staff and local key stakeholders, i.e., trained professionals and influential commune members. These (intervention) groups were called maternal and newborn health groups (MNHGs). Facilitators were recruited from the Women’s Union, a social and national governmental organization that predominantly works with issues related to women’s needs (e.g., women’s rights and sexual equality). The facilitators supported the MNHGs through monthly meetings [23]. We assumed that by including people who were already responsible for health matters, the sustainability of the approach would increase. The intention of the NeoKIP intervention was: (1) training facilitators to use a problem-solving, participatory and enabling approach; (2) empowering and supporting MNHG members to identify local problems and actions in their communes in relation to neonatal health; (3) resulting in improved health outcomes for neonates. This was a complex and multifaceted intervention, including trainers, facilitators, supervisors, MNHG members and community members. The NeoKIP trial was inspired by the Promoting Action on Research Implementation in Health Services framework, which states that successful uptake of evidence into practice is a function of context, evidence and facilitation [24]. In the NeoKIP trial, we evaluated the effect of facilitation (i.e., facilitators supporting MNHGs) on neonatal mortality, knowing that robust research evidence was available regarding best practice for neonatal care in the Vietnamese healthcare context.

Public health interventions are often implemented without evaluation of the process [25], although this can give valuable guidance while running the project and in providing explanations on its outcomes [26, 27]. According to the UK Medical Research Council [28], the key functions for a process evaluation of complex interventions are to understand: (1) the implementation of the intervention (how it is delivered and what is delivered); (2) the mechanism of impact (what are the participants’ responses to and interactions with the intervention); and (3) whether context affects the implementation and outcomes. Previously we have explored the experiences of facilitators and MNHG members regarding the facilitation intervention [29] and the influence of context [30]. In these studies, it was recognized that the MNHGs had a good mix of people and that using a coalition of a facilitator, healthcare staff and key persons was perceived to be a slow process but would have a positive impact on both MNHG members and the public if the involved stakeholders were able to collaborate. Furthermore, this type of intervention was perceived to preferably target disadvantaged groups in society. In this paper, we will present aspects of a process evaluation focusing on the implementation and the mechanisms of impact, aiding comprehension of the results of the trial, particularly the reduction on neonatal mortality in intervention communes.

## Methods

### Study setting

The NeoKIP study was conducted in Quang Ninh, a province along the north-eastern coast of Vietnam. Quang Ninh varies geographically from mountains in the inlands to plains along the coast, where there is a large archipelago with more than 2,000 islands. In some areas, transportation can be a major issue. Over the past decade, the province has shown rapid economic growth similar to the rest of Vietnam [31]. The main sources of income in Quang Ninh are coal mining industry and tourism. There are about 350,000 inhabitants living in the NeoKIP study area. Kinh is the largest ethnic group (≈85 % of the population) while the remaining population is divided into 10 minority groups. In 2005, the neonatal mortality rate was estimated to be 24 deaths per 1,000 live births [21]. Approximately 60 % of the pregnant women had three or more antenatal care visits (i.e., as recommended in the National Guidelines in Reproductive Health Care [32]); 20 % of births occurred at home.

Districts in Quang Ninh having a neonatal mortality rate ≥15/1,000 at baseline in 2005 were selected for participation in the NeoKIP study [21]. Thus, eight districts with 90 communes constituted the study area; 44 communes were randomly allocated as intervention clusters and 46 as control clusters (see Additional file 1 for the study flowchart and Additional file 2 for the CONSORT checklist). In each commune (ranging in population from 1,000 to 18,000) there is a commune health centre with three to six staff providing primary healthcare, including reproductive care. Community health workers, also called village health workers in Vietnam, are linked to the community health centre, one for each village. The community health workers are responsible for basic healthcare in the villages. Antenatal and delivery care can also be sought at any of the district hospitals, at the provincial hospital in Ha Long or at the regional hospital in Uong Bi [33]. In addition to the governmental healthcare system, there are several private alternatives for antenatal care but so far none that provides delivery services.

### Data collection and analysis

In structuring the data of the NeoKIP facilitation process, we were inspired by the framework developed by Moore et al. [28]. This framework recommends focusing a process evaluation of complex interventions on implementation, mechanism of impact and context. Thus, in this paper we will report on the implementation and the mechanisms of impact of an intervention in a Vietnamese healthcare context, while the influence of context has been reported elsewhere [29, 30].

#### Implementation

Notes from different events (pilot study, recruitments, facilitator training and meetings with various stakeholders) were used to describe the recruitment of stakeholders as well as the pilot study and the facilitator training. To capture the performance of the facilitators and MNHG members, data from different sources were collected. Written records from monthly meetings conducted with facilitators and supervisors provided information on issues raised by the facilitators, how support was performed and recurrent areas of training. Meeting times and attendance were extracted from diary entries, which facilitators recorded after their meetings with a MNHG.

#### Mechanism of impact

Information on identified problems and implemented actions was extracted from the facilitator diary. English and Vietnamese versions of the structured sections of the diary notes were entered into a Microsoft Access database. For statistical analysis, descriptive statistics (frequencies, proportions and means) were used. Health information material produced by the MNHGs to be used in dealing with the public was compared with the recommendations in the National Guidelines of Reproductive Health Care [32].

Attributes and skills of the facilitators were assessed by two of the NeoKIP researchers (DMD and NTN), who were familiar with the facilitators. The researchers individually assessed each facilitator at the end of the intervention using a scale with six items. The items included such aspects as group management skills, capacity to engage others and commitment (Table 1). For each item, the evaluators marked the response (‘Do not agree at all’ = 1, ‘Agree to some extent’ = 2, ‘Agree a great deal’ = 3 and ‘Completely agree’ = 4) that best described the facilitator that was assessed. The weighted kappa for the six items in the scale was calculated. Two methods were used to generate a score between 0 and 1 for each of the facilitators, where a higher score indicated a facilitator with greater attributes and skills. In method 1, the total number of items rating a ‘3’ or ‘4’ was divided by the total number of assessments (*n* = 12). In method 2, items rating a ‘1’ generated a score of 0, items rating a ‘2’ generated 1, items rating a ‘3’ generated 2 and items rating a ‘4’ generated 3. The scores from both researchers were added and divided by the highest possible score (*n* = 36). Both methods resulted in division of the facilitators into two identical groups, one group of facilitators that was rated ‘high’ (*n* = 7) and another group of facilitators (*n* = 4) that was rated ‘low’ (arbitrary cut-off 0.5). This result was used to group the communes into ‘high facilitator communes’ and ‘low facilitator communes’. When more than one facilitator was working in a commune during the intervention, a weighted score was calculated and used based on the time that each facilitator worked. Thereafter, the variable ‘type of commune’ was created, including ‘control communes’ (*n* = 46), ‘high facilitator communes’ (*n* = 27) and ‘low facilitator communes’ (*n* = 17). To quantify the association between the ‘type of commune’ variable (with ‘control commune’ as reference) and the outcome measure of neonatal mortality (with live births surviving the neonatal period as a reference) during the third year of intervention, a generalized linear mixed model was used. The rationale for only including the third intervention year when investigating the association between ‘type of commune’ and neonatal mortality was the timing of skills and attributes assessment (after the intervention) and the fact that it was only during the third year of the NeoKIP trial that the neonatal mortality rate differed between intervention and control communes [22]. In the models, ‘type of commune’ was treated as a fixed factor nested within the random factor commune. Results are presented by means of odds ratios and 95 % confidence intervals. For analysis, statistical software R was used [34], more specifically the packages ‘lme4’, ‘irr’ and ‘psy’ [35].

**Table 1:** Scale for assessing attributes and skills of NeoKIP facilitators

Item^a^	Description
Group management skills	The facilitator manages dynamics and processes in groups.
Communication skills	The facilitator communicates in an effective way.
Capacity to engage others	The facilitator markets the project and raises enthusiasm.
Commitment to the project	The facilitator understands and is loyal to the project.
Bravery	The facilitator dares to approach and challenge people.
Project management skills	The facilitator is skilled in organizing her job (e.g., organize meetings, supervise co-facilitators and engage in MNHG activities and monthly meetings).

^a^Items were developed with inspiration from sub-elements used in the Promoting Action on Research Implementation in Health Services framework [24] and adapted to Vietnamese conditions

### Ethical considerations

Ethical clearance was granted by the Scientific Committee of the Ministry of Health in Vietnam (Dnr 3934/QDBYT) and by the Research Ethics Committee at Uppsala University, Sweden (Dnr 2005:319). All MNHG participants gave oral consent to participate after being informed about the study. Facilitators signed a written informed consent form when they agreed to work as facilitators for the NeoKIP project.

## Results

### Implementation

Two individuals from the Women’s Union were recruited to pilot the facilitation role in 2007. They were trained for two days to gain the basic knowledge and skills required to take on this new role, followed by two days of practical work in commune groups. The training was conducted in English by two Swedish researchers (LE and LW), who are familiar with the facilitation technique, and one Vietnamese researcher (NTN), who simultaneously translated the instructions into Vietnamese. The four pilot days indicated a promising potential in having Women’s Union members as facilitators. In addition, the pilot pointed to the need to increase the length of the training and to maintain a continuous support system for the facilitators during the intervention period.

Facilitators were recruited in collaboration with the Women’s Union. Initially, local newspapers advertised the facilitator positions and then each commune was asked to suggest suitable applicants. Thereafter, the Women’s Union office in each of the eight study districts selected two individuals among the applicants for further interviews. Recruitment criteria included being an experienced Women’s Union member, having completed secondary school and having children. Hence, two NeoKIP researchers (NTN and TQH) and the chairwoman of the Women’s Union in Quang Ninh province interviewed 16 potential facilitators. Eight of the potential facilitators were selected for further training. These women were trained for 10 days by means of theoretical sessions, group discussions and role-play activities. Topics focused on during the training programme included group dynamics and quality improvement methods (e.g., brainstorming, the nominal group technique, the plan-do-study-act cycle, and the strengths-weaknesses-opportunities-threats diagnostic tool). To facilitate discussions about neonatal care, the facilitators were introduced to basic evidence-based neonatal care in accordance with the recommendations in the National Guidelines of Reproductive Health Care [32]. They were also briefed on the current health situation in the province and the function of the healthcare system in relation to reproductive health. The Swedish and Vietnamese researchers jointly developed a ‘facilitation manual’ to guide the facilitators’ daily work. This manual, which provided information on the NeoKIP facilitator role, group dynamics, different tools to use in the facilitation work and basic evidence-based neonatal care, was introduced during the facilitator training period. At the end of the training programme, the eight Women’s Union members practised their skills in rural communes outside the study area. Those practical sessions were followed up by group discussions in which researchers and co-facilitators gave feedback to individual facilitators on their performance. The training was conducted in Vietnamese by two NeoKIP researchers (TQH and NTN). Two Swedish researchers (LE and LW) were also available throughout the training period to assist when needed. The 44 intervention communes were divided between the eight facilitators based on the facilitators’ places of residence and how confident they were in the facilitator role. This division meant that each facilitator worked with five to eight MNHGs on a continuous basis.

The NeoKIP project was firmly established at different levels in the healthcare system. At joint functions, leaders from the various organizations agreed on the implementation of the intervention and were informed of the randomization outcome, i.e. which communes were randomized to be in the intervention arm and which to be in the control arm. The Provincial Health Bureau in Quang Ninh played an important role in assisting NeoKIP researchers in the process of establishing MNHGs in each of the 44 intervention communes. Each of these groups consisted of the vice chairperson of the people’s committee (i.e. the person responsible for education and health in the commune), three members of staff from the community health centre, one community health worker and a Women’s Union representative from the commune or village. In addition to these seven group members, a population collaborator (responsible for collecting population data and performing family planning) was included in the MNHG. The MNHG representatives from the village level (Women’s Union members and community health workers) were selected internally by each organization. Members selected to participate in a MNHG were encouraged to share their experiences with other members of their organizations throughout the intervention period. Meetings and actions conducted within the NeoKIP project were considered to be integrated into the MNHG members’ normal work routine and thus none of the MNHG members was paid additionally for participating in NeoKIP. Two members of the MNHG, the community health worker and the Women’s Union worker from the village level, were reimbursed their travel expenses to and from the MNHG meetings.

Of the initial eight facilitators, four worked during the whole intervention period and four left their positions after approximately 18 months because of other job opportunities (*n* = 2) or pregnancy (*n* = 2) and were replaced by three other Women’s Union members. Recruitment and training procedures were similar for all 11 facilitators. However, in connection with the training period, the three last-recruited facilitators worked alongside the four leaving facilitators for one month to become familiar with the facilitator role and introduced to the MNHGs. All facilitators who fulfilled the training programme received a contract that entitled them to a monthly salary when working as a facilitator. The 11 facilitators (nine Kinh people and two from the Tay ethnic minority group) had a mean age of 32 years at recruitment.

Two research team members (TQH and DMD) acted as supervisors of the facilitators throughout the intervention period; i.e., they supported the facilitators and assisted and coordinated the facilitation process, primarily by having monthly meetings with the facilitators and through field support. During the intervention period, 35 monthly meetings with supervisors and facilitators took place. From meeting records, we have identified the monthly meetings as an important forum for clarifying, discussing and developing the facilitator role, as well as for discussing the facilitation process and reporting on MNHGs actions. The facilitators were also continuously educated about basic evidence-based neonatal care at these support meetings, as they often requested additional information related to clinical issues and perceived such knowledge to be essential for achieving successful outcomes. Further, the facilitation diary was continuously developed to better serve as a tool for the facilitators. Supervisors and co-facilitators also attended MNHG meetings to observe and give constructive criticism to the facilitator in charge. Field support decreased over time, as the facilitators became more secure in their roles (Table 2).

**Table 2:** Data on meetings and support during the intervention period

	1st year		2nd year		3rd year		All 3 years	
	(*n*)	(%)	(*n*)	(%)	(*n*)	(%)	(*n*)	(%)
MNHG meetings	474	90	520	98	514	97	1508	95
Facilitator joined MNHG activity in a commune	68	–	166	–	60	–	294	–
Facilitator supported a co-facilitator at a MNHG meeting	102	22	11	2	9	2	122	8
NeoKIP researcher supported a facilitator at a MNHG meeting	53	11	25	5	18	4	96	6
Monthly meetings between supervisors and facilitators	11	92	12	100	12	100	35	97

The primary working tools used by the facilitators were the brainstorming technique and the plan-do-study-act cycle. A MNHG meeting with a facilitator lasted on average 110 minutes. In addition to facilitating MNHG meetings, the facilitators joined the intervention groups while implementing actions in the commune between meetings. During the implementation of actions, the facilitators took on different tasks. On some occasions, the facilitators were active in implementing an action together with the members of the MNHG while on other occasions they observed the group implementing those actions. Sometimes the facilitators assessed whether an action had been executed with the desired effect. For example, when messages were communicated at a meeting, the facilitators asked the assembled commune members if they understood and appreciated the delivered messages. Such activities occurred most frequently during the second year of the intervention (Table 2).

In total, 95 % (1508/1584) of the planned meetings with a MNHG and a facilitator were completed during the 3-year intervention (Table 2). The main reason for cancelled meetings in the first year was the difficulties in getting MNHGs organized for their first meeting. Although the intervention started in July, some MNHGs did not have their first meeting until November. After resolving this initial problem, most of the groups met regularly. In addition to the initial problem, the most common cause preventing the facilitator from assembling the MNHG during the intervention period was poor weather conditions, which mainly affected groups in mountainous and remote communes. Among the 44 MNHGs, 20 groups had the same facilitator throughout the intervention period, 22 groups changed facilitators once and 2 groups changed facilitators twice. One city-based MNHG that changed facilitators twice ceased activities in April 2010 because the group members did not believe in the project.

In total, 388 individuals served as MNHG members and participated during an average of 31 months in the NeoKIP intervention (Table 3). The overall meeting attendance among MNHG members was 86 %. Members with the highest attendance at these meetings were the head of the Women’s Union at village level, the head of the community health centre and the midwife. The vice chairperson of the peoples committee, also acting as the chair in most MNHGs, participated in approximately two-thirds of the meetings (61 %).

**Table 3:** Basic data on MNHGs and members’ attendance

Basic data	44 MNHGs
Number of participants	388
Mean time participating in the NeoKIP project (months)	31
Mean Age (years)	42
Proportion females (%)	76
Proportion belonging to Kinh group (%)	71
Attendance (%)	44 MNHGs
Overall	86
Head of Women’s Union (village level)	97
Head of community health centre	95
Midwife	94
Community health worker	90
Nurse	88
Population collaborator (commune level)	87
Chairwoman of Women’s Union (commune level)	87
Vice chairperson of peoples committee	61

### Mechanisms of impact

Altogether, the 44 MNHGs identified 32 types of problem and implemented 39 types of action (Table 4 and Additional file 3). More problems and actions were identified and implemented during the first intervention year, while the number of problems and actions declined during the second and third years. Overall, the most frequently identified problems were ‘low frequency of antenatal visits at the right time’, ‘low frequency of postnatal home visits’, ‘low awareness among pregnant women of appropriate diet, work and rest’, ‘high frequency of home deliveries’ and ‘low awareness among pregnant women about appropriate breastfeeding practices’. During the first year of the intervention, the MNHGs mostly identified problems addressing the pregnant women’s behaviour, knowledge and health (Table 4). Across the intervention period, the focus shifted such that the MNHGs were equally targeting health issues for pregnant women and for neonates.

**Table 4:** Identified problems and implemented actions among 44 maternal and newborn health groups for individual intervention years and the entire intervention period

		First year	Second year	Third year	All 3 years
Problems	Number of unique problems (*n*)	27	20	15	32
	Total number of times unique problems were identified (*n*)	151	135	94	206
	Five most commonly identified problems (*n*)^a^	Low frequency of antenatal visits at the right time (30)	Low frequency of antenatal visits at the right time (39)	Low frequency of antenatal visits at the right time (33)	Low frequency of antenatal visits at the right time (42)
		Low frequency of postnatal home visits (24)	Low frequency of postnatal home visits (28)	Low frequency of postnatal home visits (26)	Low frequency of postnatal home visits (33)
		Little awareness among pregnant women of appropriate diet regime, work and rest (20)	High frequency of home deliveries (14)	High frequency of home deliveries (14)	Little awareness among pregnant women of appropriate diet, work and rest (23)
		Little awareness among pregnant women of antenatal care (13)	Little awareness among pregnant women of appropriate diet regime, work and rest (12)	Low rate of exclusive breastfeeding (7)	High frequency of home deliveries (16)
		Little awareness among pregnant women of appropriate breastfeeding practices (10)	Low rate of exclusive breastfeeding (9)	Little awareness among pregnant women about appropriate breastfeeding practices (2) Little awareness among pregnant women about appropriate diet regime, work and rest (2) Low rate of tetanus vaccination (2)	Little awareness among pregnant women of appropriate breastfeeding practices (14)
Actions	Number of unique actions (*n*)	25	27	19	39
	Total number of times unique actions were implemented (*n*)	649	511	297	933
	Five most commonly implemented actions (*n*)^a^	Counselling at community health centre (123)	Counsel and mobilize women at their home (108)	Counsel and mobilize women at their home (72)	Counsel and mobilize women at their home (170)
		Communication at community meetings (115)	Communication at community meetings (105)	Communication at community meetings (65)	Communication at community meetings (168)
		Counsel and mobilize women at their home (108)	Counselling at community health centre (98)	Counselling at community health centre (60)	Counselling at community health centre (164)
		Communication through loudspeakers (83)	Communication through loudspeakers (61)	Postnatal home visits (41)	Communication through loudspeakers (105)
		Write communication papers (68)	Postnatal home visits (44)	Communication through loudspeakers (33)	Write communication papers (68)

^a^See Additional file 3 for a list of all problems and actions

Actions taken mainly concerned the dissemination of health information in diverse ways and forums (Table 4 and Additional file 3). The most common communication methods were ‘counselling and mobilizing women at home’, ‘communication at community meetings’, ‘counselling women at community health centres’ and ‘communicating messages through village loudspeakers’. Health information messages were produced by all 44 MNHGs, primarily compiled by the midwives in the MNHGs. The content addressed identified problems and most often adhered to the recommendations in the practice guidelines for reproductive healthcare [32]. However, these messages were sometimes on a more general level instead of giving specific guidance on how to deal with certain health problems. For instance, if low awareness of breastfeeding practices was identified as a problem, some groups would only inform mothers of the benefits of breastfeeding, whereas those providing specific information would also give instructions on how to breastfeed the child. When MNHG members communicated their messages, they used existing forums in the commune (Additional file 3). The person responsible for communication was often related to the type of meeting, i.e. a Women’s Union representative was the communicator at a Women’s Union meeting and the midwife was the communicator at the reproductive health day (Additional file 3).

The score of the 11 facilitators in the assessment of attributes and skills ranged from 0 to 1.0 (median 0.58) using method 1 and from 0.14 to 0.81 (median 0.56) using method 2. The weighted kappa for the six items in the scale ranged from 0.36 to 0.69. The neonatal mortality rate for the third year of the NeoKIP trial was lower in both ‘low facilitator communes’ (17.1/1,000) and ‘high facilitator communes’ (8.5/1,000) than in ‘control communes’ (21.1/1,000) (Table 5). However, it was only ‘high facilitator communes’ that had a significantly lower odds ratio of neonatal mortality (odds ratio, 0.37 [95 % confidence interval, 0.19–0.73]).

**Table 5:** Neonatal mortality rates and odds ratios on neonatal mortality for communes supported in ‘high facilitator’, ‘low facilitator’ or control communes during the third year in the NeoKIP trial

	Type of commune^a^		
	High facilitator commune	Low facilitator commune	Control commune
Number of communes	27	17	46
Live births	2597	1461	3695
Neonatal deaths	22	25	78
Neonatal deaths / 1,000 live births (95 % confidence interval)	8.5 (5.3–12.8)	17.1 (11.1–25.2)	21.1 (16.7–26.3)
Odds ratio (95 % confidence interval)^b^	0.37 (0.19–0.73)	0.75 (0.38–1.48)	1

^a^Type of commune includes the intervention and control communes in the NeoKIP trial. The intervention communes are stratified into two levels (high facilitator communes and low facilitator communes) based on an assessment of attributes and skills of the facilitators.

^b^Based on generalized linear mixed models with type of commune as fixed factor nested within the random factor commune and the outcome measure of neonatal mortality

## Discussion

The results from the NeoKIP trial showed that intervention clusters had a 50 % lower neonatal mortality rate than the control clusters after a latent period, i.e. during the third (and final) intervention year [22]. In this paper, we present process evaluation results focusing on the implementation of the intervention and the mechanisms of impact. A rigorous recruitment process of facilitators and MNHGs was followed by an intervention period of three years, when most of the planned facilitated MNHG meetings were performed (95 %), with a high attendance among MNHG members (86 %). The MNHGs, supported by a facilitator, identified and prioritized a wide range of problems targeting both pregnant women and their newborn children, mainly by using various types of communication activity. The attributes and skills of the facilitators seem to be contributing to the achievements of the facilitator–MNHG coalition, as the neonatal mortality rate in communes supported by facilitators who were rated high on attributes and skills was significantly less than in the control communes. In this discussion, we will elaborate on the facilitator role, the coalition between the facilitators and the MNHGs, the problems selected and the actions taken.

### The facilitator role

In the NeoKIP project, we chose to recruit facilitators without a professional healthcare background, although their role was to facilitate groups including health professionals and to maintain a focus on neonatal health. This raises the question of whether a facilitator can perform well despite being a layperson in relation to the area of implementation. During the two weeks of training, and continuously at monthly supervision meetings, the NeoKIP facilitators were provided with basic knowledge on evidence-based neonatal care. The literature is ambiguous about how much specific healthcare knowledge a facilitator actually needs [8, 9, 11, 12]. Stetler and colleagues [11] suggest that having knowledge of evidence-based practice is critical to success as a facilitator, while Harvey and co-workers [9, p. 582] suggest, ‘A mixture of personal attributes and personal, interpersonal and group management skills contribute to the development of effective facilitation.’ In a previous qualitative study [29], we found that the facilitator role in the NeoKIP intervention was sometimes questioned, particularly because of the facilitator’s lack of healthcare knowledge. Over time, the facilitators were perceived to have improved their skills and became more accepted by the MNHG members. Evidently, the intervention sites in the NeoKIP project succeeded in reducing the neonatal mortality rate substantially during the third year [22].

When comparing the participating communes, based on the assessment of the facilitators’ attributes and skills, we observed that the ‘high facilitator communes’ had lower risk of neonatal mortality during the third year of the NeoKIP trial, while the ‘low facilitator communes’ did not have significantly reduced mortality risks, as compared with control communes. This result not only suggests that it is important to consider how facilitators are trained and supported, but also how well their individual characteristics fit the facilitator role. Further, this also suggests that a facilitator can perform well despite being a layperson. A realist synthesis reviewing the literature on change agency [13] identified responsibility and accountability as essential features of successful change agents. These characteristics fit well with the items used to assess the facilitators in our study. Other features, considered to be less important in the review, included accessibility of the change agent, cultural compatibility, reflectiveness and having a positive attitude. The selection of facilitators and how to train and support them are evidently important factors to consider when initiating an intervention, especially when preparing to scale up such interventions [36]. We believe that the design of the NeoKIP intervention can be appropriate for scaling up. We used relatively few facilitators and MNHG members were recruited from existing societal systems that reach large populations. A drawback of the NeoKIP intervention might be the relatively intense involvement by supervisors from the research team. However, we anticipate that supervisors can preferably be identified within the healthcare system and be trained to function in that role.

### The facilitator–MNHG coalition

When a group is working with a facilitator, enough time for communication and relationship-building activities is needed for the group to succeed [8]. Further, key factors for successful community mobilization include allowing community members to find their own solutions, ensuring that strategies are culturally accepted and recognizing that it will take time to achieve good results [16]. Therefore, the extraordinary continuity of the NeoKIP intervention, in which 43 out of 44 MNHGs continued throughout the whole intervention period and 95 % of the intended meetings were accomplished over the 3 years, most probably contributed to the successful outcome in the third year. A joint intention of working together for common goals is fundamental for successful community coalitions [6]. Not only were the MNHG members in the NeoKIP intervention healthcare providers; they also came from other influential positions in the commune with a unique possibility of identifying local problems, mobilizing appropriate actions and implementing these actions effectively. At the same time, to have MNHGs consisting of individuals with different backgrounds might also explain why they focused on communication activities rather than actions targeting healthcare procedures. Previous studies from Canada [37] and Vietnam [38, 39] demonstrate the importance of having a leader in terms of keeping a community coalition motivated and achieving goals. The presence of a group leader has also been identified as an important factor when facilitator-led groups focus on improving practice [7, 8, 11]. A strategic decision in the NeoKIP trial was to include the vice chairperson of the people’s committee as a member of the MNHG because of that person’s decision-making position in the commune regarding education and health. Although these individuals were absent during one out of three meetings, they played an important role in the MNHGs [29, 30].

### Problems and actions

We identified certain patterns in relation to prioritized problems and actions in the MNHGs. There are probably several reasons for this. A basic feature of the MNHGs was local ownership, i.e., throughout the intervention period the MNHG members themselves decided what problems to address in their communes. This way of working is somewhat different from the *modus operandi* of previous projects using facilitators directly targeting women’s groups in low- and middle-income countries [17, 20, 40]. In those projects, the agenda was predefined; during the first year, the facilitator, together with each group, identified and prioritized problems and planned together, followed by implementation and assessment of strategies during the second year. Thus, our method was more flexible in that implementation of strategies could be performed earlier, if each MNHG continuously identified relevant problems and actions. To make this happen was a time-consuming process [29], which may explain why there was a latent period before an effect on neonatal mortality rate was shown.

Although the current trial primarily aimed at targeting problems concerning neonates, the MNHGs initially identified more problems in relation to pregnant women; ‘low frequency of antenatal visits at the right time’ was the most commonly identified problem in all three years (Table 4). A child, according to Vietnamese beliefs, is traditionally not considered a complete human being until it has passed through a number of rites [41]. One could speculate whether these perceptions are reflected in the focus on the pregnant woman rather than the delivery and the newborn child. Further, owing to a health sector reform in the 1980s, especially the richer segments of the population in large part bypass the community health centres to seek care directly at hospitals [42, 43]. This transition of primary healthcare is likely to have impacted on the MNHGs’ selection of problems in the current study, i.e. MNHGs identified fewer problems addressing the health of neonates because the community health centres were exposed to fewer neonates in their daily activities. Nevertheless, it is important to target both women and children within the whole continuum of care [1]; this was more evident in the work of the MNHGs over time.

The NeoKIP trial evaluated a complex intervention involving 44 MNHGs, each comprising eight individuals, targeting various self-selected problems by use of different combinations of actions based on each commune’s needs and resources. The groups did not have any financial support, which limited their choice of actions [29, 30] and presumably the outcome of the trial. However, we also believe this non-financed bottom-up approach to be a strong contributor to local ownership and the positive outcome of the trial.

### Methodological considerations and recommendations

A methodological limitation in this study might be that the assessment of attributes and skills of the facilitators should have been performed repeatedly during the course of the trial instead of only at the final stage. It was, however, only possible to make a well-informed assessment of these characteristics after working with the facilitators for a longer period. The facilitation element in the Promoting Action on Research Implementation in Health Services framework [24] was used in the process of developing the attributes and skills assessment scale. This scale has not yet been validated, as we developed it for the purpose of this trial. Some of the weighted kappa scores were relatively low, which could be an indication that the assessors interpreted some of the items in the scale differently. It must, however, be considered as a strength that when using two different methods for calculating scores for facilitators, they generated similar results.

A process evaluation can help explain the outcomes of an intervention [27]. However, when conducting a process evaluation without guidance there is a risk either of collecting too much data or of focusing on the wrong things. To avoid this, we propose it to be advantageous to use a framework for process evaluation when planning and designing the study; this can also incorporate the theory underpinning the implementation strategy [44]. For the purpose of this study, we used a recently published framework by Moore et al. [28] to structure the process evaluation data. This framework has been useful, but unfortunately it was not applied when originally planning the NeoKIP trial. We believe that more detailed data on how the groups actually worked using the plan-do-study-act method would have been useful, i.e. for improved understanding of how the MNHG participants prioritized problems, performed actions and how they evaluated these implemented actions. The advantage of using a process evaluation framework in a series of studies is that it will enable the gradual addition of new information regarding specific elements of the process and the making of comparisons, e.g., if evaluating the same knowledge translation strategy in different contexts. We want to underline the need of making more use of process evaluation in intervention research, guided by existing frameworks, to help researchers focus on the important issues when trying to explain the outcomes of trials.

## Conclusions

The NeoKIP trial succeeded in lowering neonatal mortality during its final (third) year by using facilitators who mobilized commune groups. In this process evaluation, we identified several factors that might have influenced the results of the trial and that are important to consider when scaling up this type intervention: to maintain a high continuity in the work of the MNHG and facilitator coalitions, to equip facilitators with adequate attributes and skills, and to focus on problems and actions that relate to the continuum of care of pregnant women and newborn children.

## Additional files

Additional file 1:
**Study flow of the NeoKIP trial.** NMR, neonatal mortality rate. (TIFF 135 kb) Additional file 2:
**CONSORT checklist.** (DOCX 38 kb) Additional file 3:
**Problems, actions and different kinds of meetings.** Unique problems and actions identified in all 44 maternal and newborn health groups for the entire intervention period and a list of different kinds of meetings where communication activities were conducted. (DOCX 116 kb)

## Abbreviations

MNHG: maternal and newborn health group
NeoKIP: Neonatal Health – Knowledge Into Practice
