# Supplementary material for: Process evaluation of a knowledge translation intervention using facilitation of local stakeholder groups to improve neonatal survival in the Quang Ninh province, Vietnam
Source: Trials. 2016 Jan 13;17:23. doi: 10.1186/s13063-015-1141-z (PMC4711002; doi:10.1186/s13063-015-1141-z)
Supplement: Additional file 3: — Problems, actions and different kinds of meetings. Unique problems and actions identified in all 44 maternal and newborn health groups for the entire intervention period and a list of different kinds of meetings where communication activities were conducted. (DOCX 116 kb) [file 13063_2015_1141_MOESM3_ESM.docx]

### Problems, actions and different kinds of meetings

### Identified unique problems and actions in all 44 Maternal and Newborn Health Groups (MNHGs) for the entire intervention period and a list of different kinds of meetings where communication activities were conducted.

| **Types of problem (number of times occurring)** |
| --- |
| 1. Low frequency of antenatal care visits at the right time (42) 2. Low frequency of post-natal home visits (33) 3. Low awareness among pregnant women of appropriate diet, work and rest (23) 4. High frequency of home deliveries (16) 5. Low awareness among pregnant women of appropriate breast feeding practices (14) 6. Low awareness among pregnant women of antenatal, delivery and neonatal care (13) 7. Low awareness among pregnant women of tetanus vaccination (10) 8. Low awareness among pregnant women of dangerous symptoms during pregnancy (10) 9. Low frequency of women who exclusively breastfeed (10) 10. Low awareness among pregnant women of prenatal and neonatal care (4) 11. High frequency of women who have >3 children (4) 12. Low awareness among pregnant women of the benefits of iron supplementation (3) 13. High rate of neonatal mortality (2) 14. Low coverage of tetanus vaccination (2) 15. Low quality of pre-, delivery- and post-natal care and management (2) 16. Low awareness among pregnant women of delivering at health facilities (2) 17. Low awareness among pregnant women of appropriate neonatal care practices (1) 18. Low awareness among pregnant women of family planning (1) 19. Low awareness among pregnant women of diarrhoea treatment for neonates (1) 20. Low awareness among pregnant women of prevention of low-birth weight (1) 21. Low awareness among pregnant women of gynaecological diseases (1) 22. Low awareness among pregnant women of reproductive health care issues (1) 23. Pregnant women do not adhere to drug prescription (1) 24. Hard work during period of pregnancy (1) 25. High frequency of <18-year-old women that miscarriage (1) 26. High rate of stillbirths (1) 27. Negative environmental impacts on pregnant women (1) 28. Pregnant women are at risk of coming into contact with toxic material (1) 29. High frequency of gynaecological diseases of women in reproductive age (1) 30. Too many preterm births (1) 31. Pregnant women are not found early in their pregnancy and therefore lack antenatal care (1) 32. Low quality of foetus monitoring (1) |
| **Types of action (number of times occurring)** |
| 1. Counsel and mobilise women at their home (170) 2. Communication at community meetings***** (168) 3. Counselling at community health centres (CHCs) (164) 4. Communication through loudspeakers (105) 5. Write communication papers (68) 6. Supply knowledge to community health workers (73) 7. Post-natal home visits (63) 8. Distribute leaflets about pre- and post-natal care (24) 9. Developing lists of pregnant women and women who have child(ren) <1 year (14) 10. No activity (14) 11. Communicate messages to women in villages (6) 12. Estimate number of deliveries to plan for home visit (5) 13. Mobilise and communicate to family of pregnant women (4) 14. Evaluate activity implementation (4) 15. MNHG members supervise community health workers at their assigned areas (3) 16. Train community health workers about antenatal care / family planning (3) 17. Developing lists of mothers who exclusively breast feed for an overview (3) 18. Communicate messages to small groups in villages (3) 19. Monitor foetus and pregnant woman (weight, circumference, etc.) (3) 20. Distribute iron tablets (3) 21. Developing lists of women in their reproductive age for overview (2) 22. Group counselling of women in villages (2) 23. Invite women with risky pregnancy for examination (2) 24. Examine pregnant women at CHC (2) 25. Assign role to report for next meeting (2) 26. Distribute communication papers to community health worker (2) 27. Developing lists of postpartum women for overview (1) 28. Developing lists of post-natal home visits for overview (1) 29. Developing lists of adolescents for overview (1) 30. Developing lists of women who have child(ren) < 6 months old for overview (1) 31. Distribute communication paper to pregnant women (1) 32. Population collaborator to monitor pregnant women (1) 33. Divide a specific village into areas to overcome home deliveries (1) 34. Find pregnant women early to mobilise them for antenatal care (1) 35. Find reasons for home delivery (1) 36. Head of MNHG asks for money support for poor pregnant women in the commune (1) 37. Improve quality of labour care (1) 38. Health staff perform post-natal care for abnormal women (1) 39. Distribute sterilised delivery kits (1) |
| ***Different kinds of forums where communication was conducted** |
| 1. Women’s Union meeting – arranged quarterly in every village where Women’s Union members are invited to become involved in various discussions. 2. Nutrition day – arranged by nurse/midwife 2-3 times per year in targeted areas of the commune to inform and discuss about nutrition with the population 3. Reproductive health day - arranged by a nurse/midwife 2-3 times per year regarding targeted areas in the community to inform and discuss reproductive health with the population 4. Sympathy club – this club is part of the Women’s Union and the participants help poor and vulnerable in society 5. Dutch non-governmental organisation based in Tien Yen district since 2008 targeting poor and vulnerable communes in various ways to establish affordable and **quality health care** 6. Immunization day – arranged every month at the CHC for pregnant women and small children to get antenatal care check-up and immunization 7. Population campaign – arranged 1-2 days per year by the National Committee for Population, Family and Children to inform the population about nutrition and family planning 8. Poverty Eradication Program – a program targeting the poor in society run by the Women’s Union 9. Children Care Programs at CHC targeting various areas 10. Village meeting – gatherings of all heads of households in each village 1-2 times per ye 11. Women’s Groups in villages – arranged because of the *Neonatal health – Knowledge Into Practice project* |
